# Dense U-net Based on Patch-Based Learning for Retinal Vessel Segmentation

**DOI:** 10.3390/e21020168

**Published:** 2019-02-12

**Authors:** Chang Wang, Zongya Zhao, Qiongqiong Ren, Yongtao Xu, Yi Yu

**Affiliations:** 1 School of Biomedical Engineering, Xinxiang Medical University, Xinxiang City Engineering Technology Research Center of Neurosensor and Control, Xinxiang 453003, China; 2 School of Biomedical Engineering, Xinxiang Medical University, Xinxiang Key Laboratory of Biomedical Information Research; Henan Engineering Laboratory of Combinatorial Technique for Clinical and Biomedical Big Data, Xinxiang 453003, China

**Keywords:** Retinal vessel segmentation, Dense U-net, convolutional neural network, patch-based learning strategy, data augmentation

## Abstract

Various retinal vessel segmentation methods based on convolutional neural networks were proposed recently, and Dense U-net as a new semantic segmentation network was successfully applied to scene segmentation. Retinal vessel is tiny, and the features of retinal vessel can be learned effectively by the patch-based learning strategy. In this study, we proposed a new retinal vessel segmentation framework based on Dense U-net and the patch-based learning strategy. In the process of training, training patches were obtained by random extraction strategy, Dense U-net was adopted as a training network, and random transformation was used as a data augmentation strategy. In the process of testing, test images were divided into image patches, test patches were predicted by training model, and the segmentation result can be reconstructed by overlapping-patches sequential reconstruction strategy. This proposed method was applied to public datasets DRIVE and STARE, and retinal vessel segmentation was performed. Sensitivity (Se), specificity (Sp), accuracy (Acc), and area under each curve (AUC) were adopted as evaluation metrics to verify the effectiveness of proposed method. Compared with state-of-the-art methods including the unsupervised, supervised, and convolutional neural network (CNN) methods, the result demonstrated that our approach is competitive in these evaluation metrics. This method can obtain a better segmentation result than specialists, and has clinical application value.

## 1. Introduction

Retinal vessel segmentation has a great clinical application value for diagnosing hypertension, arteriosclerosis, cardiovascular disease, glaucoma, and diabetic retinopathy [1]. Various retinal vessel segmentation methods have been proposed recently, and these methods can be categorized as unsupervised and supervised approaches according to whether the manually labeled ground truth is used or not.

For the unsupervised methods, multi-scale enhanced-vessel filtering, multi-threshold vessel detection, matched filtering, morphological transformations, and model-based algorithms are predominant. The entropy of some particular antennas with a pre-fractal shape, harmonic sierpinski gasket and weierstrass-mandelbrot fractal function were studied, and the result indicated that their entropy is linked with the fractal geometrical shape and physical performance [2,3,4]. Multi-scale enhanced-vessel filtering using second order local structure feature was proposed, and the vessel and vessel-like pattern was enhanced by Frangi et al. in 1998 [5]. Three-dimensional (3D) multi-scale line filter was applied to the segmentation of brain vessel, bronchi, and liver vessel by Sato et al. in 1998 [6]. A general vessel segmentation framework based on adaptive local threshold, the automatic determining of the local optimal threshold by the verification-based multi-threshold probing strategy, and the retinal vessel segmentation were completed by Jiang et al. in 2003 [7]. A locally adaptive derivative filter was designed, and filter-based segmentation method was proposed for retinal vessel segmentation by Zhang et al. in 2016 [8]. A combination of shifted filter responses (COSFIRE) operator was used to detect retinal vessel or vessel-like pattern, the improved COSFIRE was designed and applied to the segmentation of retinal vessel by Azzopardi et al. in 2015 [9]. A new infinite parameter active contour model with hybrid region information was designed, and it was applied to the segmentation of retinal vessel by Zhao et al. in 2015 [10]. Level set method based on regional energy-fitting information and shape priori probability was proposed to segment blood vessel by Liang et al. in 2018 [11]. The unsupervised methods always design filters that are sensitive to vessel and vessel-like patterns, and it will lead to blood vessels not being fully identified and wrongly identified vessel-like pseudo patterns. The unsupervised methods depended on parameters settings; unsuitable parameters settings will produce low-quality segmentation results.

For the supervised methods, firstly, the features of retinal vessel were selected and extracted. Secondly, ground truth was used to train the classifier. Lastly, retinal vessels were identified by use of a classifier. The features a of retinal vessel can be extracted by Gabor transform, discrete wavelet transform [12,13], vessel filtering, Gaussian filtering, and so on. Traditional machine learning methods such as k-nearest neighbor, adaboost, random forest, and support vector machine were used to train the classifier [14]. Orlando et al. proposed a fully connected conditional random field model, using a structured output support vector machine to learn model parameters, and performed retinal vessel segmentation [15]. Zhang et al. extracted the features by vessel filtering and wavelet transform strategy, applied the random forest training strategy learn the classifier’s parameters, and performed retinal vessel segmentation [16]. For the traditional machine learning methods, feature selection has great influence on segmentation accuracy, and the independent features with high vessel recognition rate is the critical step. The features need to be selected manually according to the experiment; feature automatic selection remains a hot topic.

Convolutional neural networks (CNNs) have drawn more and more attention, since they can automatically learn complex hierarchies of features from input data [17]. CNNs were widely applied to image classification, recognition, and segmentation [18]. Fully convolutional networks (FCN) as semantic segmentation network were proposed by Long et al., including designing the skip architecture that combines semantic information for a deep, coarse layer with appearance information from a shallow; the semantic segmentation task was completed by FCN [19]. The U-net model was proposed by Ronneberger et al. in 2015 [20], which designed a contracting path and an expansive path which combined captured context with precise localization; this model was successfully applied to biomedical image segmentation. However, the public dataset for retinal vessel is limited. U-net cannot achieve perfect vessel segmentation results using the training and prediction strategy based on the entire image. Brancati et al. divided the retinal vessel images into patches, proposed a U-net based on patch-based learning strategy, and achieved a perfect segmentation result by [21]. A multi-scale fully convolutional neural network was proposed to cope with the varying width and direction of vessel structure in the fundus images, and then the stationary wavelet transform was used to provide multi-scale analysis; the rotation was used as data augmentation and retinal vessel segmentation was performed by Oliveira et al. [22]. A novel reinforcement sample learning scheme was proposed to train CNN with fewer iterations of epochs and less training time; retinal vessel segmentation was performed by Guo et al. in 2018 [23]. A retinal vessel segmentation method based on convolutional neural network (CNN) and fully connected conditional random fields (CRFs) was proposed by Hu et al. in 2018 [24], and an improved cross-entropy loss function was designed to solve the class-unbalance problem. The densely connected convolutional network (DenseNet) [25,26] and Inception-ResNet [27] were proposed in the past two years. Dense block can encourage feature reuse and alleviate the vanishing-gradient problem; the layers are directly connected with all of their preceding layers within each dense block. DenseNet utilized dense blocks and improved classification performance. Dense U-net as semantic segmentation network was proposed and applied to scene segmentation by Jégou S. et al. in 2017 [28]. In their study, the fully connect layers of DenseNet were dropped and the skip architecture was used to combines semantic information for a deep, coarse layer with appearance information from a shallow.

Inspired by the fact that U-net can improve segmentation accuracy of retinal vessel by the patch-based training and testing strategy, we proposed a new retinal vessel segmentation framework based on Dense U-net and patch-based learning strategy. In this segmentation framework, retinal vessel images were divided into image patches as training data by random extraction strategy. Dense U-net was used as network model, and the model parameters were learned by training data. In this model, loss function based on dice coefficient was designed, and was optimized by stochastic gradient descent (SGD). The proposed method was applied to public datasets DRIVE and STARE, and retinal vessel segmentation was performed. Sensitivity (Se), specificity (Sp), accuracy (Acc), and area under each curve (AUC) were adopted as evaluation metrics to verify the effectiveness of proposed method. Compared with state-of-the-art methods including the unsupervised, supervised, and CNN methods, the result demonstrated that the proposed method is competitive in these evaluation metrics.

The contributions of our work were elaborated as follows:

(1) We proposed the retinal vessel segmentation framework based on Dense U-net and patch-based learning strategy.

(2) Random transformation was used as data augmentation strategy to improve the network generalization ability.

The rest of this paper is organized as follows: Section 2 presents the proposed method; Section 3 analyzes and discusses the experiment result; Section 4 concludes this study.

## 2. Method

**Figure 1:**
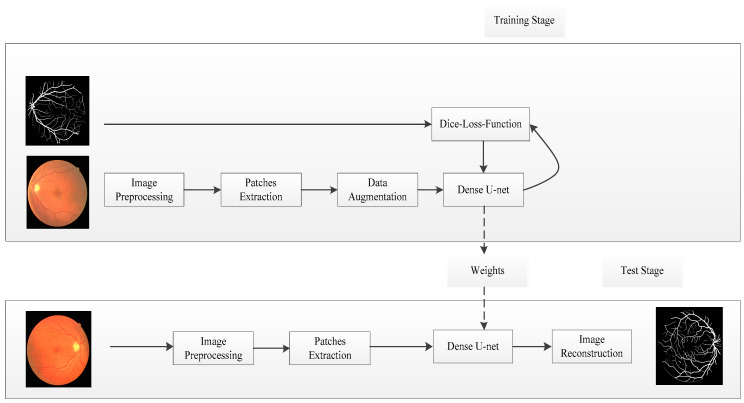
Overview of the proposed method.

In this study, we proposed the retinal vessel segmentation framework based on Dense U-net and patch-based learning strategy. This framework was shown in Figure 1; it contains training and testing in two stages.

In the training stage, the source retinal vessel image was converted into a gray image, and data normalization was used as an image preprocessing strategy. The image patches can be obtained as training data by random extraction strategy. Dense U-net was used as network model, loss function based on dice coefficient was optimized by stochastic gradient descent (SGD), and the model weight parameters were learned by training data.

In the test stage, the test images were processed by the same preprocessing strategy, test patches were obtained by overlapping extraction strategy, and the segmentation results were obtained by overlapping-patches sequential reconstruction strategy.

### 2.1. Patches Extraction

For fundus images, the retinal vessel manual segmentation was both error-prone and time consuming, and ground truth of the retinal vessel was limited. In our approach, patch-based learning strategy was used in the process of training and testing. The training and labeled patches were extracted from training and labeled images by random extraction strategy, respectively, and these patches were used as training data to train model parameters. The testing patches were extracted from testing images by overlapping extraction strategy, and the predicted result was reconstructed by overlapping-patches sequential reconstruction strategy.

**Figure 2:**
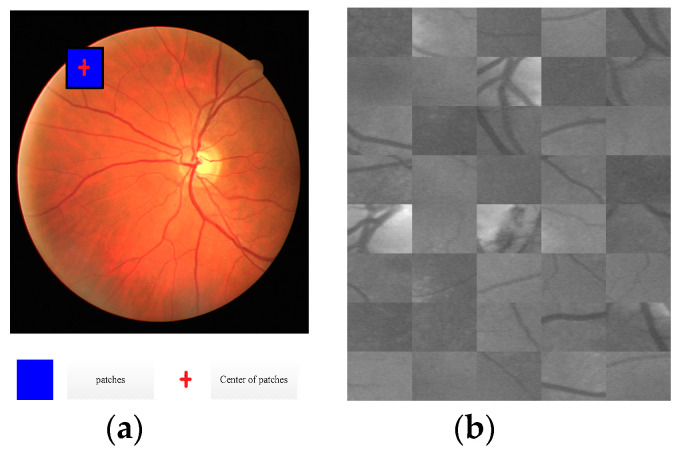
Patches extraction (**a**) patches extraction strategy (**b**) patches extraction result.

In the process of training, the patches were extracted randomly from training images, and the number of patches for each image was the same. Randomly extracted strategy was described by Algorithm 1. The judging strategy that the central coordinates of image patch was inside the field of view (FOV) is shown in Figure 2a, and the randomly extracted image patches are shown in Figure 2b.

**Algorithm 1.** Image patches random extraction strategy   ***Input***: Source image, ground truth   ***Output***: Patches_source and patches_ground    Calculate patches number ***N_patch_per_image*** for each image basing on the principle of equal distribution    ***for*** i=1 to ***N_images***
     k=0     ***while*** k< ***N_patch_per_image***       generate the central coordinates of patch randomly       judge the central coordinates of image patch inside FOV       produce patches_source and patches_ground   k=k+1    ***return*** patches_source and patches_ground

In the process of testing, each testing image was divided into several testing patches by overlapping extraction strategy and the number of testing patches for each image were calculated with Equation (1):

(1) $$N\_patches\_per\_img=(\left\lfloor \frac{img\_h-patch\_h}{stride\_h}\right\rfloor +1)\times (\left\lfloor \frac{img\_w-patch\_w}{stride\_w}\right\rfloor +1)$$

where img_h and img_w are the size of source image, patch_h and patch_w are the size of extracted patch, stride_h and stride_w are stride length, and the operator ⌊⌋ is rounded down to the nearest integer.

The overlapping extracted patches were predicted by the training model, the retinal vessel segmentation result was reconstructed by overlapping-patches sequential reconstruction strategy, and the reconstruction strategy is described by Algorithm 2.

**Algorithm 2.** Overlapping-patches sequential reconstruction strategy   ***Input***: Patch-base prediction result ***preds***, image size ***img_h***, ***img_w***, stride ***stride_h***, ***strid_w***   ***Output***: Final segmentation result ***final_avg***    Calculate patches number ***N_patches_h*** in height for each image    Calculate patches number in width ***N_patches_w*** for each image    Calculate patches number ***N_patches_img*** for each image    ***for*** i=1 to ***N_patches_img***
     ***for*** h=1 to ***N_patches_h***       ***for*** w=1 to ***N_patches_w.***
         obtain pixel predicted probability ***full_pro***
         obtain pixel predicted frequency ***full_sum***    Calculate final segmentation result ***final_avg***    ***return*** final segmentation result

In Algorithm 2, ***N_patches_h***, ***N_patches_w*, *N_patches_img*** were calculated by Equations (2)–(4):

(2) $$N\_patches\_h=\left\lfloor \frac{img\_h-patch\_h}{stride\_h}\right\rfloor +1$$

(3) $$N\_patches\_w=\left\lfloor \frac{img\_w-patch\_w}{stride\_w}\right\rfloor +1$$

(4) N_patches_img=N_patches_h×N_patches_w

where ***img_h*** and ***img_w*** are the size of image and ***stride_h*** and ***stride_w*** are stride length. ***full_pro*** and ***full_sum*** are the probability and frequency summation of pixels that belonged to image patches, and the image patches were obtained by overlapping extraction strategy. ***final_avg*** as the final segmentation result was calculated with Equation (5):

(5) $$final\_avg=\frac{full\_pro}{full\_sum}$$

### 2.2. Dense U-net Architecture

Convolutional neural networks can learn the higher-level features from the characteristics of the lower-level layer, and then drop the low-level features. The low re-use rate of features cannot effectively improve the network’s learning ability, thus, improving the utilization rate of features is more significant than adding the depth of networks. In order to improve the utilization rate of features, a dense block was designed, and the layers were directly connected with all of their preceding layers within each dense block. DenseNet improved classification performance using dense block. DenseNet was extended to fully convolutional networks for semantic segmentation named as Dense U-net, which was applied to scene segmentation. However, the retinal blood vessel is tiny: the width of a blood vessel is multi-pixel or even single-pixel. The features of retinal blood vessels can be learned effectively by using a patch-based learning strategy, and the segmentation accuracy of a retinal vessel by U-net depending on patch-based learning strategy is higher than U-net. Thus, Dense U-net using the patch-based learning strategy was proposed as a retinal vessel segmentation framework.

**Figure 3:**
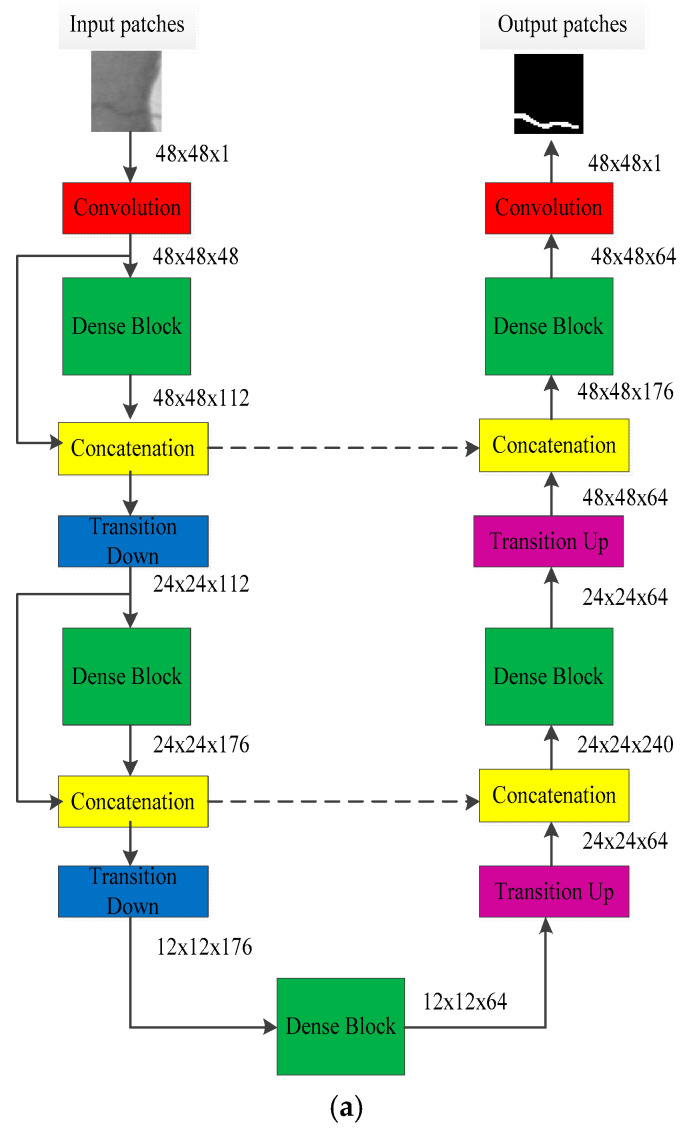

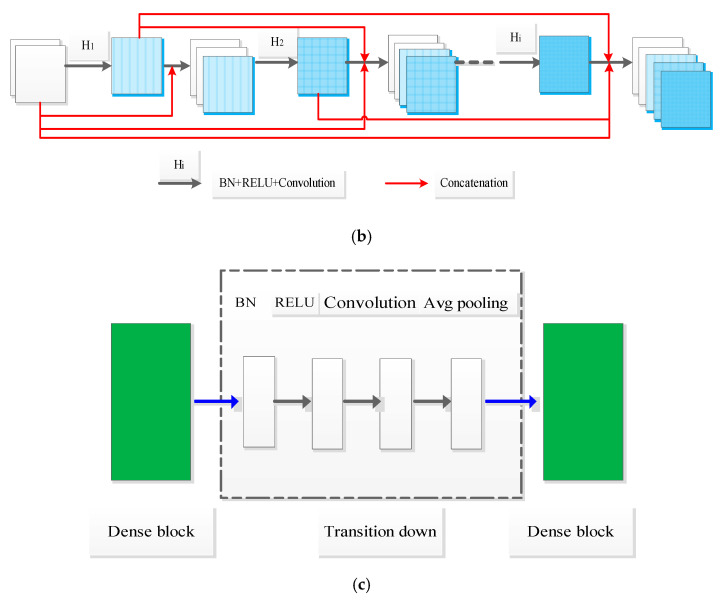
Patches training for semantic segmentation. (**a**) Dense U-Net architecture; (**b**) dense block; (**c**) transition down.

Dense U-net was used as training network, and it is shown in Figure 3a. The randomly extracted image patches were used as training data, whose resolution is 48×48. The model output is the predicted result, and it represents vessel segmentation result. Dense U-net consists of a contracting path (left side) and an expansive path (right side); it contains dense block, a transition layer, and concatenation.

#### 2.2.1. Dense Block

In traditional CNN, the output of $l^{\mathrm{th}}$ layer can be calculated by a non-linear transformation strategy, which is defined by Equation (6):

(6) $$x_{l}=H_{l}(x_{l-1})$$

where $x_{l}$ is the output of $l^{\mathrm{th}}$ layer, $x_{l-1}$ is the output of $(l-1{)}^{\mathrm{th}}$ layer, and H is defined as a convolution followed by rectified linear unit (ReLU) and dropout.

In order to reuse the previous features, ResNets [24] designed residual block, which adds a skip-connection that bypasses the non-linear transformations, and it is defined by Equation (7):

(7) $$x_{l}=H_{l}(x_{l-1})+x_{l-1}$$

where H is defined as the repetition of a residual block, consisting of batch normalization (BN), followed by ReLU and a convolution.

DenseNet [20] designed dense block, which can use all of preceding features in a feedforward fashion, defined by Equation (8):

(8) $$x_{l}=H_{l}(\left[x_{0},x_{1},\ldots ,x_{l-1}\right])$$

where […] represents the concatenation operation and $H_{i}$ is defined as a composite function that consists of three consecutive operations: batch normalization (BN), followed by a rectified linear unit (ReLU) and a 3 × 3 convolution (Conv). The dense block is shown in Figure 3b, and it has l layers. Dense block strongly encourages the reuse of features and makes all layers in the architecture receive a direct supervision signal. It will produce k feature-maps by transition function for each layer; k is defined as the growth rate of network. Suppose that the channel of feature maps in input layer is $k_{0}$, the channel of output feature maps will be $k_{0}+k\times (l-1)$. Growth rate can regulate the contribution of new information for each layer to the global feature maps.

#### 2.2.2. Transition Layer

The layers between dense blocks were named transition layers, and contain transitions down and transitions up. Transition down layer is defined in Figure 3c, and it consists of these consecutive operations: BN, followed by a ReLU, a 1 × 1 Conv, and a 2 × 2 average pooling for down sampling. The transition up layer was implemented by a 2 × 2 up sampling.

### 2.3. Loss Function

The pixels can be categorized into vessel and non-vessel; the statistical result indicates that only 10% of the pixels were retinal vessels for the fundus image. The vessel and non-vessel pixels are highly imbalanced ratio [29]. If it was not considered in the process of designing loss function, the learning process would be inclined to segment non-vascular region. The learning process will be trapped in the local minima of loss function, and the vessel pixels are often lost or only partially identified.

Loss function based on class-balanced cross-entropy was proposed by Xie et al. [30]; however, the loss value is influenced by the weight coefficient. In our approach, a novel loss function based on the dice coefficient [31] was adopted, ranging from 0 and 1. The dice coefficient can be defined by Equation (9):

(9) $$D=\frac{2\sum_{i}^{N}p_{i}g_{i}}{\sum_{i}^{N}p_{i}{}^{2}+\sum_{i}^{N}g_{i}{}^{2}}$$

where N is the number of label pixels, $p_{i}$ and $g_{i}$ are the predicted result and ground truth, respectively. This formulation can be differentiated yielding the gradient as follows:

(10) $$\frac{\partial D}{\partial p_{j}}=2\left[\frac{g_{j}(\sum_{i}^{N}p_{i}{}^{2}+\sum_{i}^{N}g_{i}{}^{2})-2p_{j}(\sum_{i}^{N}p_{i}g_{i})}{(\sum_{i}^{N}p_{i}{}^{2}+\sum_{i}^{N}g_{i}{}^{2}{)}^{2}}\right]$$

### 2.4. Data Augmentation and Preprocessing

In data preprocessing, the training image (RGB) was converted into grayscale. Data normalization strategy was utilized, defined by Equation (11):

(11) $$X^{*}=\frac{X-\mu }{\sigma }$$

where X and $X^{*}$ are grayscale image and normalization image and μ and σ are the mean value and standard deviation of all training images, respectively.

**Figure 4:**
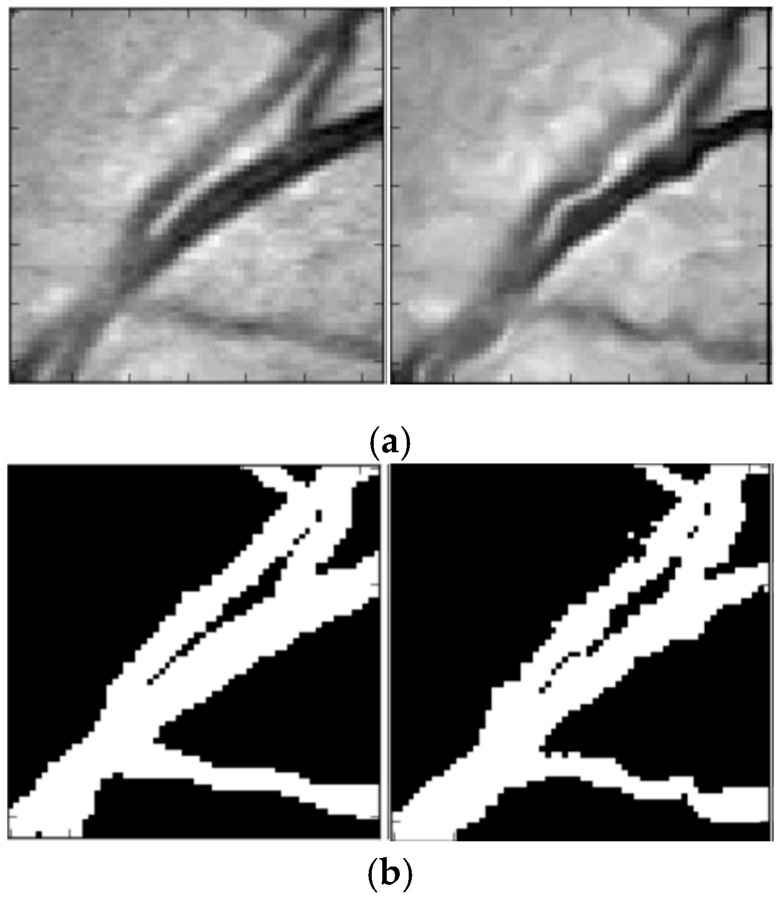
Data augmentation result. (**a**) Image patches; (**b**) corresponding ground truths.

In the process of retinal vessel segmentation, convolutional neural network methods can easily fall into overfitting [32]. Data augmentation was used to increase the training sets and improve the generalization ability of network model. In our approach, the resolution of extracted patches was 48 × 48, and the patches were used as the input of dense u-net. Non-linear transformation as data augmentation strategy was proposed by Simard [33], which was created by uniformly generating a random transformation field, defined by U(x,y)=rand(−1,+1). The data augmentation result is shown in Figure 4. Figure 4a is the source image patches, and Figure 4b is ground truth. The left is the original patch, and the right is the augmented patch.

## 3. Result

### 3.1. Experiment

#### 3.1.1. Experiment Data

The public datasets DRIVE [34] and STARE [35] were used to demonstrate the effectiveness of the proposed method. The DRIVE database contains training and testing sets. The training set contains source image, mask image, and ground truth; there were 20 source images, (RGB) whose resolution was 565 × 584. In the process of training, the image patches as training set were extracted from source images by using a random extraction strategy; the number of extracted patches was 40000, whose resolution was 48 × 48. A cross-validation strategy was utilized, and 10% of the training data was used as the validation set. In the process of testing, the testing images were divided into test patches by overlapping extraction strategy, and the extracted patches were used as a testing set. The parameters, using the overlapping extraction strategy, were set as follows: stride_height = 5 and stride_width = 5. The final segmentation result can be reconstructed by the overlapping-patches sequential reconstruction strategy. Two specialists manually segmented the testing images; the segmentation result by the first specialist was used as ground truth, the second was used as the gold standard of the first manual result. The STARE dataset also contains 20 images whose resolution is 700 × 605: the images were divided into 10 training and 10 testing image in order to validate the effectiveness of the proposed method. In the process of training and testing, the patch-based learning strategy and parameter setting were the same.

All experiments were conducted on a Linux Mint 18 OS server, equipped with Intel Xeon Gold 6130 CPU, NVIDIA TITAN X GPU, 12 GB of RAM. Dense U-net was used as the network model and the parameters were set as follows: number of epoch = 150, growth rate = 16, number of dense block = 2, layers of each dense block = 5. SGD (learning rate = 0.01, momentum = 0.9) was selected as the optimization function of network model. Training time by proposed method was 2 h, and memory was 880 M.

#### 3.1.2. Evaluation Metrics

There were four kinds of segmentation results including true positive (TP), false negative (FN), true negative (TN), and false positive (FP), based on the fact that each pixel can be segmented correctly or incorrectly. Four indicators were utilized as evaluation metrics, which can be defined as follows: Sensitivity (Se), Specificity (Sp), Accuracy (Acc), and the area under each curve (AUC). The first three indicators can reflect the segmentation ability of vessel pixels, non-vessel pixels, and all the pixels, respectively.

(12) $$\mathrm{Se}=\frac{\mathrm{TP}}{\mathrm{TP}+\mathrm{FN}}$$

(13) $$\mathrm{Sp}=\frac{\mathrm{TN}}{\mathrm{TN}+\mathrm{FP}}$$

(14) $$\mathrm{Acc}=\frac{\mathrm{TP}+\mathrm{TN}}{\mathrm{TP}+\mathrm{FN}+\mathrm{TN}+\mathrm{FP}}$$

AUC, which represent the area under the ROC curve, was also adopted as an evaluation metric for image segmentation, ranging from 0 to 1.

### 3.2. Validation of the Proposed Method

**Figure 5:**
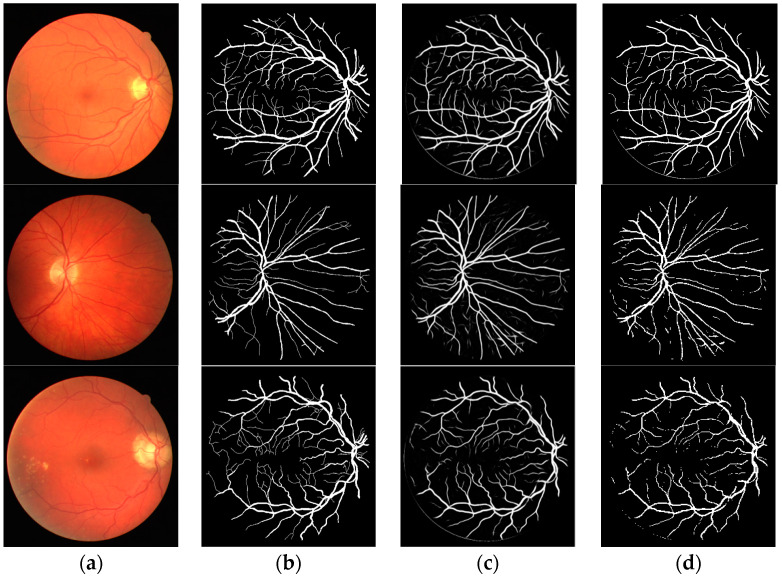
Segmentation results of DRIVE by the proposed method. (**a**) Color fundus image; (**b**) ground truth; (**c**) probability map; (**d**) binarization result.

**Figure 6:**
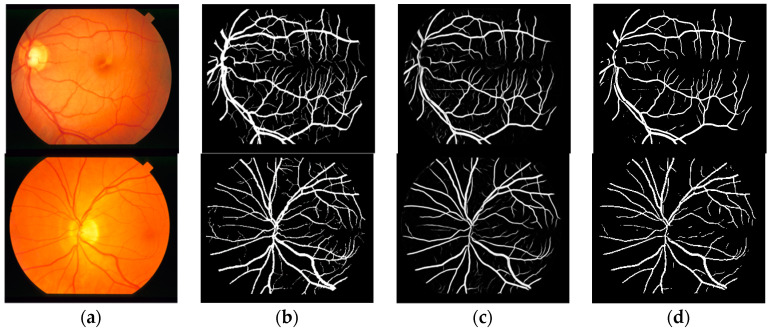
Segmentation results of STARE by the proposed method. (**a**) Color fundus image; (**b**) ground truth; (**c**) probability map; (**d**) binarization result.

The effectiveness of the proposed method was demonstrated by public data DRIVE and STARE; the segmentation result of proposed method with dice loss function is shown in Figure 5 and Figure 6. Figure 5 is the segmentation result for public data DRIVE; Figure 5a–d are color fundus image (test image), ground truth (specialist manual segmentation result), probability map for retinal vessel by proposed method, and binarization of probability map, respectively. Figure 6 is the segmentation result for public data STARE. The result demonstrated that retinal vessel segmentation can be performed with the proposed method.

Se, Sp, Acc, and AUC were utilized as evaluation metrics for the segmentation result. Random transformation field was adopted as a data augmentation strategy; the base of the proposed method is that 40000 real patches were extracted as a training set. The base and augmented data were that 40000 real extracted patches and 40000 augmented patches were used as a training set; the statistical result is shown in Table 1. The segmentation result of the second observer was used as the gold standard. The result showed that Acc and AUC for segmentation on the DRIVE dataset increased from 0.9483 and 0.9686 to 0.9511 and 0.9740, respectively. Acc and AUC for the segmentation on the STARE dataset increased from 0.9508 and 0.9684 to 0.9538 and 0.9704, respectively. Random transformation field as a data augmentation strategy can improve the ability of retinal vessel identification.

### 3.3. Comparison with U-net

Retinal vessel segmentation result by the proposed method was compared with the U-net based on patch-based learning strategy. In the contrast experiments, the image patches extraction strategy by these two methods was the same. In the process of training, the random extraction strategy was used to obtain training set, and in the process of testing, overlapping extraction and overlapping-patches sequential reconstruction strategies were used to obtain the final segmentation result. In the process of training, the number of extracted patches (40000) was the same for these two methods; 40000 augmented patches by random transformation field were produced, and 80000 image patches were used as training data. In order to evaluate these two methods fairly, the depth of two training networks = 3. SGD (learning rate = 0.01, momentum = 0.9) was selected as the optimization function by U-net, which was the same as proposed method. Because dense block strongly encourages the reuse of features and makes all layers in the architecture receive direct supervision signal, it has more parameters than the ‘standard’ U-net. More convolution layers were used at the same resolution by U-net to make sure that the numbers of trainable parameters by these two methods were approximately equal. In general, a fair comparison was made to evaluate these two methods.

**Figure 7:**
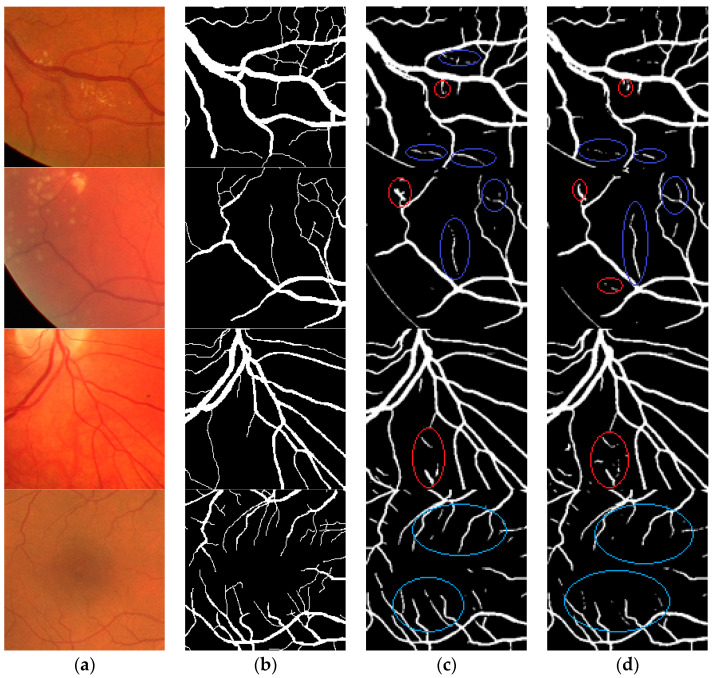
Comparison of segmentation result by Dense U-net and U-net. (**a**) The local region of fundus images; (**b**) ground truth; (**c**) binarization results by Dense U-net with dice loss function; (**d**) binarization results by U-net with dice loss function.

Figure 7 displays the local segmentation results by Dense U-net and U-net with dice loss function, respectively. The blue area is the segmentation result of fine retinal vessel, and the red area is the error segmentation result. The results demonstrated that more fine blood vessels can by segmented by the proposed method, and that a more accurate region prone to leakage and error segmentation can be segmented by the proposed method.

The quantitative analysis based on evaluation metrics for public data DRIVE and STARE is shown in Table 2. For these two methods, the values of Se, Acc, and AUC using the dice loss function were higher than those when using the cross entropy loss function. Only Sp was lower when using the dice loss function compared to using the cross entropy loss function. This demonstrates that the segmentation accuracy using dice loss function was higher than that using cross entropy function.

The result showed that for public DRIVE and STARE data, Se increased from 0.7937 and 0.7882 to 0.7986 and 0.7914, respectively. For Sp, Acc, and AUC values were approximately equal for these two methods using the dice loss function.

### 3.4. Comparison with the State-of-the-art Methods

The proposed method was compared with other state-of-the-art approaches, including unsupervised, supervised, and convolutional neural networks methods on public DRIVE and STARE datasets. The statistical result is shown in Table 3. Acc and AUC values were 0.9511 and 0.9740, respectively, for the DRIVE dataset and Acc and AUC values were 0.9538 and 0.9704, respectively for the STARE dataset by the proposed method.

For the multi-scale convolutional neural network method proposed by Hu et al. [17], Sp and AUC value are the highest. In their study, Hu proposed an improved cross entropy loss function that was influenced by the weight coefficient, and applied CRFs as a post processing strategy to get the final binarization segmentation result. Their segmentation result was influenced by the weight coefficient, and this parameter needs to be set manually. For convolution neural network method with reinforcement sample learning strategy proposed by Guo et al. [18], the Se value was the highest, Sp and Acc value was the lowest, and the final segmentation result was the worst. For U-net based on patch-based learning strategy, Se, Sp, Acc and AUC value were not the highest; however, the segmentation result was the best in the comprehensive evaluation. For the proposed method, the Se value was higher than that of U-net, and Sp, Acc, and AUC value were close to U-net. This means that the segmentation result of proposed method is similar to U-net, and that the recognition rate of blood vessel is higher than U-net. Se, Sp, Acc, and AUC values by the proposed method were higher than the specialist result, which demonstrates that the segmentation result by the proposed method is better than specialist results, and that this method has clinical application value.

## 4. Conclusions

In this study, retinal vessel segmentation framework based on patch-based learning strategy and Dense U-net was proposed. The random extraction strategy was used to obtain image patches as training data, the Dense U-net was adopted as training network model, and the dice loss function was optimized by stochastic gradient descent (SGD). Random transformation field was used as a data augmentation strategy to enlarge the training data and improve the generalization ability. The proposed method was applied to public datasets DRIVE and STARE to complete the retinal vessel segmentation. Se, Sp, Acc, and AUC were adopted as evaluation metrics to demonstrate the effectiveness of the proposed method. The results demonstrated that the proposed method is competitive in these evaluation metrics. The segmentation accuracy by proposed method was higher than that of specialist, showing that this method has clinical application value. There is no post-processing strategy in this study, and the breakage of fine blood vessels was produced in the process of binarization. Therefore, post-processing strategy may also improve our results in future work.

## Figures and Tables

**Table 1:** Performance of proposed method on DRIVE and STARE.

	DRIVE				STARE			
Proposed method	Se	Sp	Acc	AUC	Se	Sp	Acc	AUC
Second human observer	0.7760	0.9724	0.9472		0.8952	0.9384	0.9349	
40000 real	0.7886	0.9716	0.9483	0.9686	0.7904	0.9716	0.9508	0.9684
40000 real +40000 augmented	0.7986	0.9736	0.9511	0.9740	0.7914	0.9722	0.9538	0.9704

Se: sensitivity; Sp: specificity; Acc: accuracy; AUC: the area under each curve

**Table 2:** The performance of the proposed method in DRIVE and STARE.

	DRIVE				STARE			
Method	Se	Sp	Acc	AUC	Se	Sp	Acc	AUC
Second human observer	0.7760	0.9724	0.9472		0.8952	0.9384	0.9349	
U-net (dice-loss)	0.7937	0.9747	0.9517	0.9745	0.7882	0.9729	0.9547	0.9740
U-net (cross-entropy)	0.7758	0.9755	0.9500	0.9742	0.7838	0.9780	0.9535	0.9673
Dense U-net (dice-loss)	0.7986	0.9736	0.9511	0.9740	0.7914	0.9722	0.9538	0.9704
Dense U-net (cross-entropy)	0.7886	0.9736	0.9483	0.9716	0.7896	0.9734	0.9475	0.9682

**Table 3:** The performance of proposed method on DRIVE and STARE.

		DRIVE				STARE			
Type	Method	Se	Sp	Acc	AUC	Se	Sp	Acc	AUC
	Second expert observer	0.7760	0.9724	0.9472		0.8952	0.9384	0.9349	
Unsupervised	Zhao [7]	0.7420	0.9820	0.9540	0.8620	0.7800	0.9780	0.9560	0.9673
	Azzopardi [6]	0.7655	0.9704	0.9442	0.9614	0.7716	0.9701	0.9497	0.9563
	Zhang [5]	0.7743	0.9725	0.9776	0.9636	0.7791	0.9758	0.9554	0.9748
Supervised	Orlando [10]	0.7897	0.9684	0.9454	0.9506	0.7680	0.9738	0.9519	0.9570
	Zhang [11]	0.7861	0.9712	0.9466	0.9703	0.7882	0.9729	0.9547	0.9740
Deep learning	Hu [19]	0.7772	0.9793	0.9533	0.9759	0.7543	0.9814	0.9632	0.9751
	Guo [18]	0.8990	0.9283	0.9199	0.9652				
	U-net	0.7937	0.9747	0.9517	0.9745	0.7882	0.9729	0.9547	0.9740
	Our proposed	**0.7986**	**0.9736**	**0.9511**	**0.9740**	**0.7914**	**0.9722**	**0.9538**	**0.9704**
